# Identification of a Role for the PI3K/AKT/mTOR Signaling Pathway in Innate Immune Cells

**DOI:** 10.1371/journal.pone.0094496

**Published:** 2014-04-09

**Authors:** Songbo Xie, Miao Chen, Bing Yan, Xianfei He, Xiwen Chen, Dengwen Li

**Affiliations:** State Key Laboratory of Medicinal Chemical Biology, College of Life Sciences, Nankai University, Tianjin, China; French National Centre for Scientific Research, France

## Abstract

The innate immune system is the first line of host defense against infection and involves several different cell types. Here we investigated the role of the phosphatidylinositol 3 kinase (PI3K) signaling pathway in innate immune cells. By blocking this pathway with pharmacological inhibitors, we found that the production of proinflammatory cytokines was drastically suppressed in monocytes and macrophages. Further study revealed that the suppression was mainly related to the mammalian target of rapamycin (mTOR)/p70^S6K^ signaling. In addition, we found that the PI3K pathway was involved in macrophage motility and neovascularization. Our data provide a rationale that inhibition of the PI3K signaling pathway could be an attractive approach for the management of inflammatory disorders.

## Introduction

Innate immune cells, comprised of monocytes, leukocytes, and macrophages, behave as the first line of defense against infection and respond to pathogens immediately by recruiting immune cells to the loci of infection, following the identification and clearance of the pathogens [1]. During the processes, macrophages along with dendritic cells, act as antigen presenting cells in the context of major histocompatibility complex by exposing the foreign antigen to T lymphocytes to initiate primary immune responses [2]. Macrophages are derived from monocytes, which circulate in the blood and when tissues are damaged or infected, these monocytes migrate into the affected tissue where they differentiate into tissue resident macrophages [3], [4]. The tissue macrophages display anatomical difference and have diverse functions, including development, metabolic homeostasis, tissue repair, and immune responses to pathogens [5]. Due to the significance of macrophages in normal physiology and development, they are thought to have a crucial role in inflammatory defense and also cause the pathogenesis of inflammatory disorders [6]. Moreover, the inflammatory macrophages produce a number of proinflammatory cytokines/chemokines, which activate the defense mechanism through transforming cytolytic cells to effector cells to eliminate foreign substance [7]. However, emerging evidence reveals that the inflammatory macrophages are also involved in chronic inflammatory and autoimmune diseases in spite of their benefit of clearance of pathogen at the beginning [8].

Proinflammatory cytokines, mainly released by immune cells, play a critical role in immune response as well as development and differentiation of immune cells. In contrast, a dysregluated cytokine release contributes to inflammatory diseases, such as systemic lupus,rheumatoid arthritis, multiple sclerosis, atherosclerosis, and diabetes, as well as immune escape of cancerous cells [9]–[11]. Some proinflammatory cytokines, such as interleukin-1β (IL-1β), interleukin-6 (IL-6), interleukin-8 (IL-8), and tumor necrosis factor α (TNF-α), which usually are termed as ‘bad guys’, are reported to correlate with inflammation response and immune regulation as well as several immune diseases [12]. For example, the secretion of IL-1β, IL-6, and TNF-α from macrophages results in metabolic disease and insulin resistance [13], [14]. Investigation of the secretion of cytokines and their function in host is beneficial for illuminating the mechanisms of pathogenic development and exploring effective therapeutic strategies for inflammatory disorders.

PI3K was first discovered as an oncogenic gene, which transforms normal cells to tumor cells in vitro [15], [16]. PI3K, classified into three classes according to its structure and function, functions through phosphorylating phosphatidylinositol (4,5)-bisphosphate (PIP2) on the inositol ring 3′-OH position to generate the second messenger phosphatidylinositol (3,4,5)-trisphosphate (PIP3), which subsequently activates protein kinase B (PKB/AKT) and mTOR through a cascade of signal transduction [17], [18]. PI3K signaling is involved in a diversity of cellular behaviors, including proliferation, survival, metabolism, trafficking, and immunity [19]. Recent data indicate that inhibition of PI3K signaling could attenuate immune responses by suppression of secretion of proinflammatory cytokines [20], which provides the rationale that intervention of PI3K pathway could be an effective strategy for inflammatory associated disorders, such as neurodegenerative disorders, cardiovascular diseases, autoimmune diseases, and tumorigenesis [21]. Therefore, there is increasing demand for uncovering the mechanism of PI3K signaling in immunity. In the present study, we analyze several cytokines simultaneously using multiple approaches. Our data reveal that inhibition of PI3K signaling decreases the production of proinflammatory cytokines IL-1β, IL-6, IL-8, TNF-α, granulocyte colony-stimulating factor (G-CSF), and vascular endothelial growth factor (VEGF). Furthermore, we evaluate the effect of these cytokines on the motility of macrophages, as well as neovascularization. These findings suggest a potential of pharmacological inhibition of the PI3K pathway for the treatment of inflammatory disorders.

## Materials and Methods

### Cell Culture

HUVECs, Jurkat T cells, and THP-1 cells were purchased from ATCC. HUVECs and Jurkat T cells were maintained in the RPMI-1640 medium supplemented with 10% FBS. THP-1 cells were cultured in the RPMI-1640 medium supplemented with 10% FBS, 0.05 mM 2-mercaptoethanol. The differentiation of THP-1 monocytes to macrophages was performed by addition of 50 ng/ml phorbol-12-myristate-13-acetate (Sigma-Aldrich, St. Louis, MO) for 48 hours. For time course study, THP-1 cells and THP-1 derived macrophages were treated with lipopolysaccharides (LPS) (Sigma-Aldrich, St. Louis, MO) and the supernatants of media were collected at 2, 4, and 6 hours, respectively. For cytokine determination, THP-1 cells and THP-1 derived macrophages were treated with LY294002 (LY) (Sigma-Aldrich, St. Louis, MO) for 3 hours and then stimulated with LPS for 6 hours. Supernatants of conditioned media (CM) were collected for Luminex and MSD based multiplex analyses. Likewise, conditioned media were also used for cell motility and neovascularization assays.

### Luminex Based Multiplex Cytokine Detection

The assay was performed according to the manufacturer’s manual. Briefly, the panel of magnetic xMAP microsphere beads conjugated with G-CSF, IL-6, TNF-α, and VEGF antibodies (Millipore, Billerica, MA) were mixed together and transferred into 96-well plates and washed with PBS plus 1% BSA twice by evacuation of liquid through Luminex magnetic plate separator. Then cell supernatants or serially diluted standards were added to the indicated wells. After 2 hours of incubation, the plate was washed with PBS plus 1% BSA for three times. Following biotinylated detection antibodies were added to each well. After 1 hour of incubation, the plate was washed with PBS plus 1% BSA twice, and subsequently Streptavidin-R-phycoerythrin Conjugate (SAPE) were added and incubated for 30 minutes. After final wash, the xMAP microsphere beads were suspended in sheath liquid. The data collected from Luminex 200 were analyzed and the amount of cytokines was calculated by comparing with the standard curve.

### Whole Blood Donation

Whole blood were collected from healthy volunteer and used under their written informed consent for MSD based multiplex analysis for cytokine determination. The use of human samples was approved by the Medical Ethics Committee of Nankai University.

### MSD based Multiplex Cytokine Detection

The assay was performed according to the manufacturer’s manual. In brief, MSD multi-spot plate precoated with IL-1β, IL-6, IL-8, and TNF-α antibodies (Meso Scale Discovery, Rockville, MD) was blocked with 2.5% BSA for 1 hour, and then incubated with cell supernatants or serially diluted standards for 2 hours. After wash with PBS plus 0.05% Tween, SULFO-TAG labeled detection antibody was added and incubated for 1 hour. Then the plate was washed for three times and T read buffer was added for detection. Data acquired from Sector Imager 6000 were analyzed and the amount of cytokines was determined by comparing with the standard curve.

### Phosphorylation Analysis

Jurkat T cells and THP-1 derived macrophages were treated with PI3K inhibitors LY or Wortmannin (Sigma-Aldrich, St. Louis, MO), mTOR inhibitors AZD8055 (AZD) (Santa Cruz Biotechnology, USA) or Rapamycin (Sigma-Aldrich, St. Louis, MO), respectively. Then cells were lyzed with phosphosafe extraction buffer (Millipore, Billerica, MA) and the phosphorylation level of Akt, GSK-3β, and p70^S6K^ was analyzed by Akt Signaling Whole Cell Lysate Kit (Meso Scale Discovery, Rockville, MD). In brief, the plate was blocked with 2.5% BSA for 1 hour and then incubated with cell lysates for 2 hours. The plate was washed with PBS plus 0.05% Tween, and SULFO-TAG labeled detection antibody was added and incubated for 1 hour. The plate was then washed twice with PBS plus 0.05% Tween and T read buffer was then added. Data were analyzed with Sector Imager 6000 instrument.

### Transwell Assays

Cell motility was carried out by transwell assays as described previously [22]. Briefly, THP-1 derived macrophages were suspended in serum free medium and then plated into insert (Corning, USA) precoated with matrigel (BD Biosciences, USA). The lower chambers were filled with conditioned media from THP-1 derived macrophages, which were treated with LPS with/without inhibitors, respectively. IL-1β, IL-6, IL-8, TNF-α, G-CSF, or VEGF (Sigma-Aldrich, St. Louis, MO) was added to the lower chamber of the indicated wells. Cells on the inner side of the inserts were wiped 16 hours later, and cells on the underside of the inserts were stained with crystal violet. The photograph was taken under the inverted microscope.

### Trans-endothelial Cell Migration Assay

HUVECs were seeded on FluoroBlok insert (BD Biosciences, USA) precoated with fibronectin. Two days after HUVECs were confluent, THP-1 derived macrophages were harvested and labeled with 5 μg/ml DiIC12(3) fluorescent dye (BD Biosciences, USA) for 45 minutes. Then cells were plated onto HUVECs. Conditioned media from THP-1 derived macrophages were added to the lower chamber. IL-1β, IL-6, IL-8, TNF-α, G-CSF, or VEGF was added to lower chamber of the indicated wells, respectively. After incubation for 16 hours, the migrated macrophages were photographed under the inverted fluorescent microscope, and measured using the bottom read pattern at excitation/emission, 485/530 nm.

### Adhesion Assay

For cell-ECM adhesion assay, the 96-well plate was coated with 50 ng/ml fibronectin at 4°C overnight, and for cell-endothelium adhesion assay, the 96-well plate was seeded with HUVECs to form endothelial monolayer. THP-1 derived macrophages were labeled with calcein-AM prior to assay. Then cells were suspended in different conditioned media and seeded onto fibronectin or HUVEC monolayer. Cells were washed 30 minutes later with PBS to remove the non-adherent cells. Adherent cells were measured at excitation/emission, 485/530 nm.

### Tube Formation Assay

Neovascularization was performed as described previously [23] with minor modification. In brief, 50 μl matrigel was added to 96-well plates and incubated at 37°C for 30 minutes to allow matrigel to solidify. HUVECs resuspended in different conditioned media were plated onto matrigel and incubated at 37°C with 5% CO_2_ for 16 hours. Then calcein-AM was added to label HUVECs. Photographs were taken and overall tube length was analyzed with Image J pro software.

### Statistical Analysis

All data were derived from three independent experiments, and presented as means ± SD. Student’s t-test and one-way analysis of variance (ANOVA) were performed for statistical analysis. P value <0.05 was considered statistically significant.

## Results

### Inhibition of PI3K Suppresses the Production of Proinflammatory Cytokines Secreted by Immune Cells

**Figure 1:**
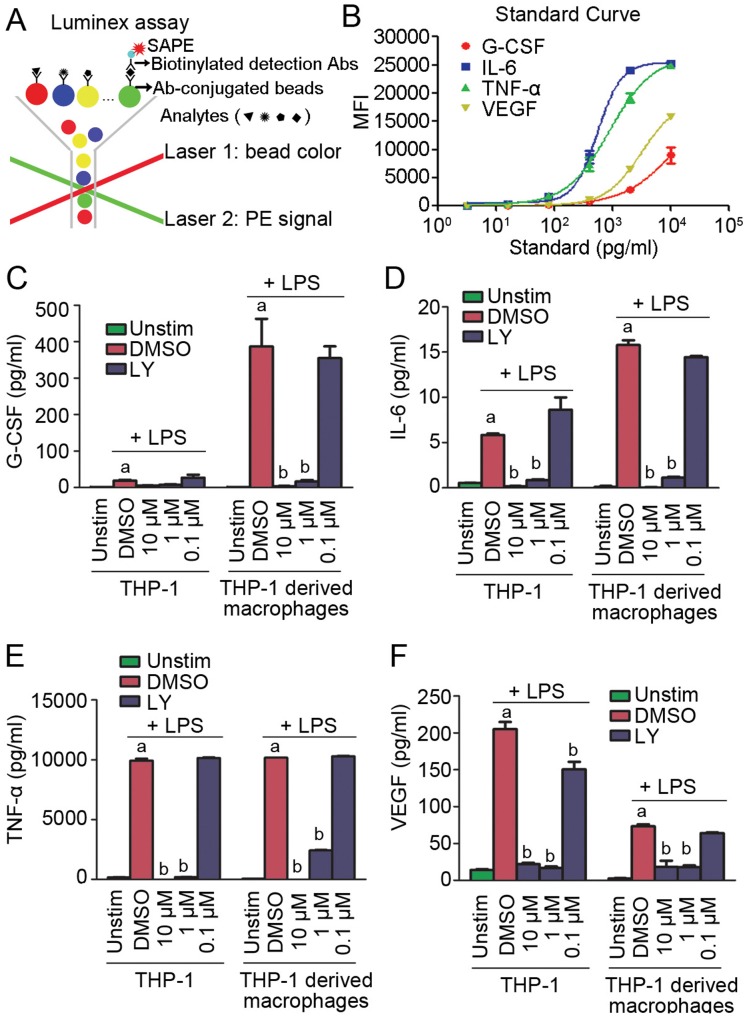
The PI3K inhibitor LY suppresses the secretion of proinflammatory cytokines from monocytes and macrophages. (A) Diagram of Luminex based multiplex assays. Antibodies against cytokines were conjugated with different color-coded beads to capture the analytes, and biotinylated detection antibodies and Streptavidin R-Phycoerythrin Conjugate (SAPE) were then added sequentially to bind the captured analytes. The mixture was analyzed with Luminex 200 instrument, whereby the bead set is distinguished by a red laser and the PE signal on the bead is determined by a green laser. (B) Standard curve determination of G-CSF, IL-6, TNF-α, and VEGF. (C–F) Analysis of proinflammatory cytokines from THP-1 monocytes and THP-1 derived macrophages. a indicates P<0.05 versus Unstim. b indicates P<0.05 versus DMSO.

To investigate the role of PI3K in immune cells, we analyzed the production of proinflammatory cytokines by blocking the activity of PI3K using the pan-PI3K inhibitor LY. We performed Luminex based multiplex assays to analyze simultaneously the secretion of G-CSF, IL-6, TNF-α, and VEGF. As shown in Figure 1A, the different color-coded microspheres were distinguished by a red laser, and meanwhile the assay signal was detected by a green laser. The standard curve was obtained with serially diluted standards to determine the amount of each cytokines (Figure 1B). Firstly we performed a time-course study to determine the optimal time for LPS treatment. G-CSF, IL-6, TNF-α, and VEGF labeled xMAP microspheres were incubated with supernatants collected 2, 4, and 6 hours of LPS treatment, respectively, and the production was determined with the Luminex 200 instrument. G-CSF level was elevated markedly after 6 hours of LPS stimulation in THP-1 derived macrophages (Figure S1 A). The production of IL-6 and TNF-α was significantly increased after LPS treatment and reached the maximum at 6 hours in both THP-1 monocytes and THP-1 derived macrophages (Figure S1 B & C). Although the production of VEGF was lower in THP-1 derived macrophages compared to THP-1 monocytes, much stronger induction of VEGF secretion was observed after LPS treatment at 6 hours (Figure S1 D). We thus adopted the time period of 6 hours for LPS treatment in the following assays. THP-1 monocytes and THP-1 derived macrophages were treated with LY for 3 hours, and then cells were stimulated with LPS for 6 hours. The supernatants were analyzed using the Luminex assay. LPS significantly induces the release of G-CSF, IL-6, TNF-α, and VEGF in both THP-1 monocytes and THP-1 derived macrophages; however, the induction effect was inhibited by LY in a dose-dependent manner (Figure 1C–F).

**Figure 2:**
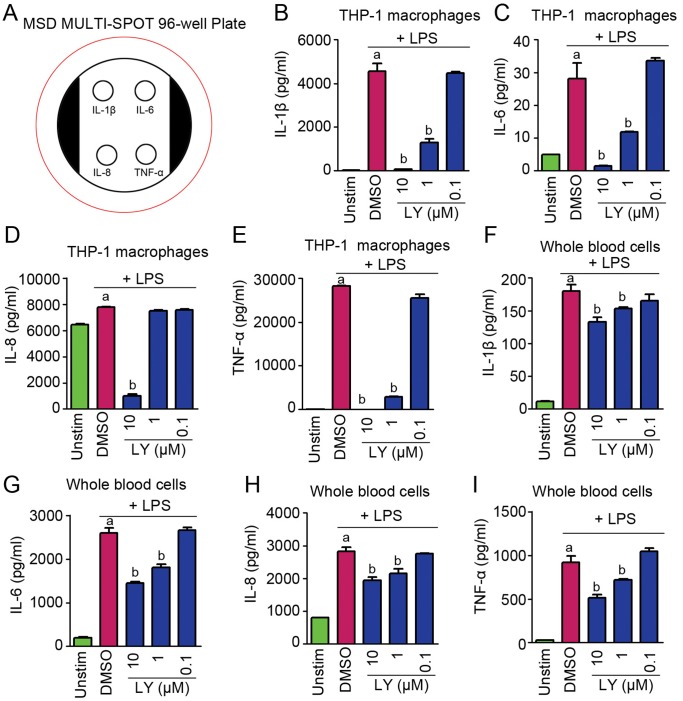
The PI3K inhibitor LY suppresses the secretion of proinflammatory cytokines from macrophages and whole blood cells. (A) Diagram of multiplex plate spots precoated with analyte capture antibodies. (B–E) Determination of production of IL-1β, IL-6, IL-8, and TNF-α from THP-1 derived macrophages. (F–I) Determination of production of IL-1β, IL-6, IL-8, and TNF-α from whole blood cells. a indicates P<0.05 versus Unstim. b indicates P<0.05 versus DMSO.

Subsequently, we examined the effect of PI3K signaling on other proinflammatory cytokines using MSD based multiplex assays. IL-6 and TNF-α were re-analyzed and used as reference controls in MSD assays to make the data comparable with those obtained from Luminex assays. Proinflammatory cytokines from THP-1 derived macrophages were examined by using the multi-spot MSD plate precoated with IL-1β, IL-6, IL-8, and TNF-α antibodies (Figure 2A). As shown in Figure 2B–E, LPS remarkably promotes the secretion of IL-1β, IL-6, IL-8, and TNF-α in THP-1 derived macrophages, whereas its promotion was abolished by LY in a dose-dependent manner. To confirm the results obtained in monocytes and macrophages, we investigated the production of these cytokines in whole blood, which encompasses different immune cell types. Similarly, LY impeded the secretion of cytokines induced by LPS stimulation (Figure 2F–I). Taken together, these data suggest that the production of proinflammatory cytokines is orchestrated by PI3K signaling in immune cells, especially in macrophages.

### PI3K and mTOR Inhibitors Significantly Influence the Phosphorylation of Akt and p70^S6K^ and only Slightly Affect GSK-3β

**Figure 3:**
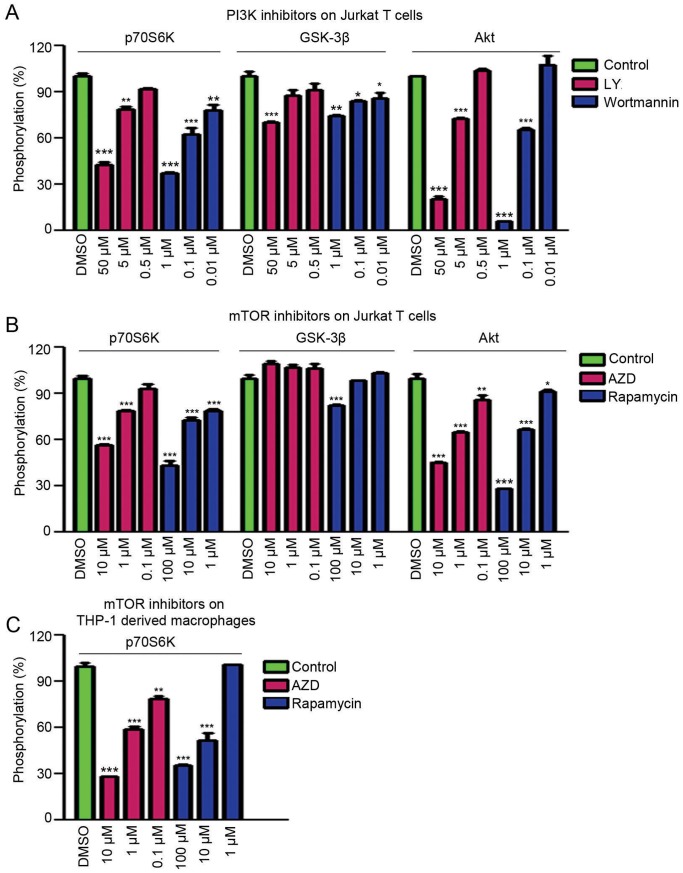
Effects of PI3K and mTOR inhibitors on phosphorylation of downstream effectors. (A) Analysis of the phosphorylation level of Akt, GSK-3β, and p70^S6K^ in LY or Wortmannin treated Jurkat T cells. (B) Analysis of the phosphorylation level of Akt, GSK-3β, and p70^S6K^ in AZD or Rapamycin treated Jurkat T cells. (C) Determination of the phosphorylation level of p70^S6K^ in AZD or Rapamycin treated THP-1 macrophages. *, **, and *** indicate P<0.05, P<0.01, P<0.001 versus DMSO, respectively.

The PI3K signaling is involved in the secretion of proinflammatory cytokines, but the molecular mechanism remains unclear. To elucidate the mechanism of which effectors are involved in PI3K mediated cytokine secretion, we analyzed the phosphorylation of its downstream effectors Akt, GSK-3β, and p70^S6K^ with MSD based multiplex assays. PI3K inhibitors LY and Wortmannin, or mTOR inhibitors AZD and Rapamycin were used to block the PI3K signaling in Jurkat T cells, in which Akt is constitutively active because of the deficiency of phosphatase and tensin homolog (PTEN). Multi-spot MSD plates precoated with phospho-p70^S6K^ (Thr421/Ser424), phospho-GSK-3β (Ser9), and phospho-Akt (Ser473) antibodies were incubated with cell lysates treated with PI3K inhibitors or mTOR inhibitors, and phosphorylated Akt, GSK-3β, and p70^S6K^ were assessed by MSD assays. As shown in Figure 3A, PI3K inhibitors LY and Wortmannin suppressed the phosphorylation of Akt and p70^S6K^ in a dose-dependent manner, whereas both of these two inhibitors only show slight effect on GSK-3β. Next, we investigated whether the inhibition of PI3K is mediated through the PI3K/Akt/mTOR axis. By treating Jurkat T cells with AZD or Rapamycin, we found that the phosphorylation of p70^S6K^ was decreased in a dose-response manner. In addition, phosphorylated Akt was also suppressed by mTOR inhibitors, which is supposed to result from the effect of mTORC2 on Akt. Similarly, phosphorylated GSK-3β was slightly affected by AZD or Rapamycin (Figure 3B). Furthermore, the involvement of PI3K signaling in macrophages was also investigated. THP-1 derived macrophages were treated with the PI3K inhibitor LY or the mTOR inhibitor AZD to determine the phosphorylation of p70^S6K^. Interestingly, p70^S6K^ is remarkably regulated by both inhibitors (Figure 3C). Collectively, these data reveal that inhibitors targeting PI3K/Akt/mTOR pathway restrain the production of proinflammatory cytokines, probably through their action on the activity of mTOR/p70^S6K^.

### Inhibition of mTOR Activity Decreases the Production of Proinflammatory Cytokines from Macrophages

**Figure 4:**
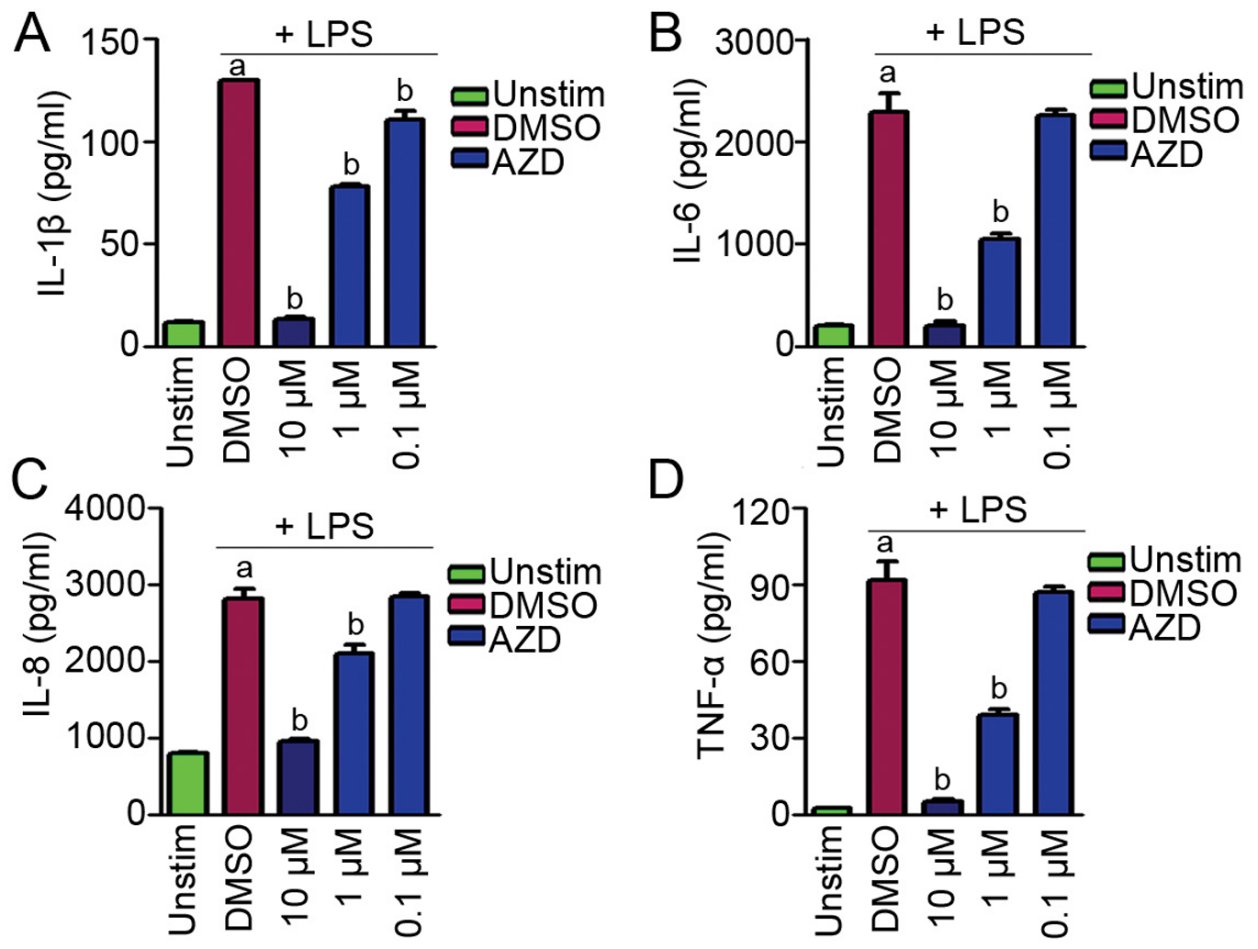
The mTOR inhibitor AZD decreases the expression of IL-1β, IL-6, IL-8, and TNF-α from THP-1 derived macrophages. Assessment of the secretion of IL-1β, IL-6, IL-8, and TNF-α in AZD treated THP-1 macrophages. a indicates P<0.05 versus Unstim. b indicates P<0.05 versus DMSO.

To examine whether the secretion of proinflammatory cytokines is also affected by mTOR inhibitors, we analyzed the production of IL-1β, IL-6, IL-8, and TNF-α using MSD based multiplex assays. THP-1 derived macrophages were treated with the mTOR inhibitor AZD for 3 hours, and then LPS was added to stimulate the release of cytokines. Supernatants were collected 6 hours later and assayed in multi-spot MSD plates precoated with IL-1β, IL-6, IL-8, and TNF-α antibodies. As shown in Figure 4A–D, LPS enhances the production of IL-1β, IL-6, IL-8, and TNF-α, whereas the mTOR inhibitor AZD antagonizes the effect of LPS stimulation in a dose-dependent manner.

### The Motility and Adhesion of Macrophages are Mediated by the PI3K Signaling through its Action on Cytokine Secretion

**Figure 5:**
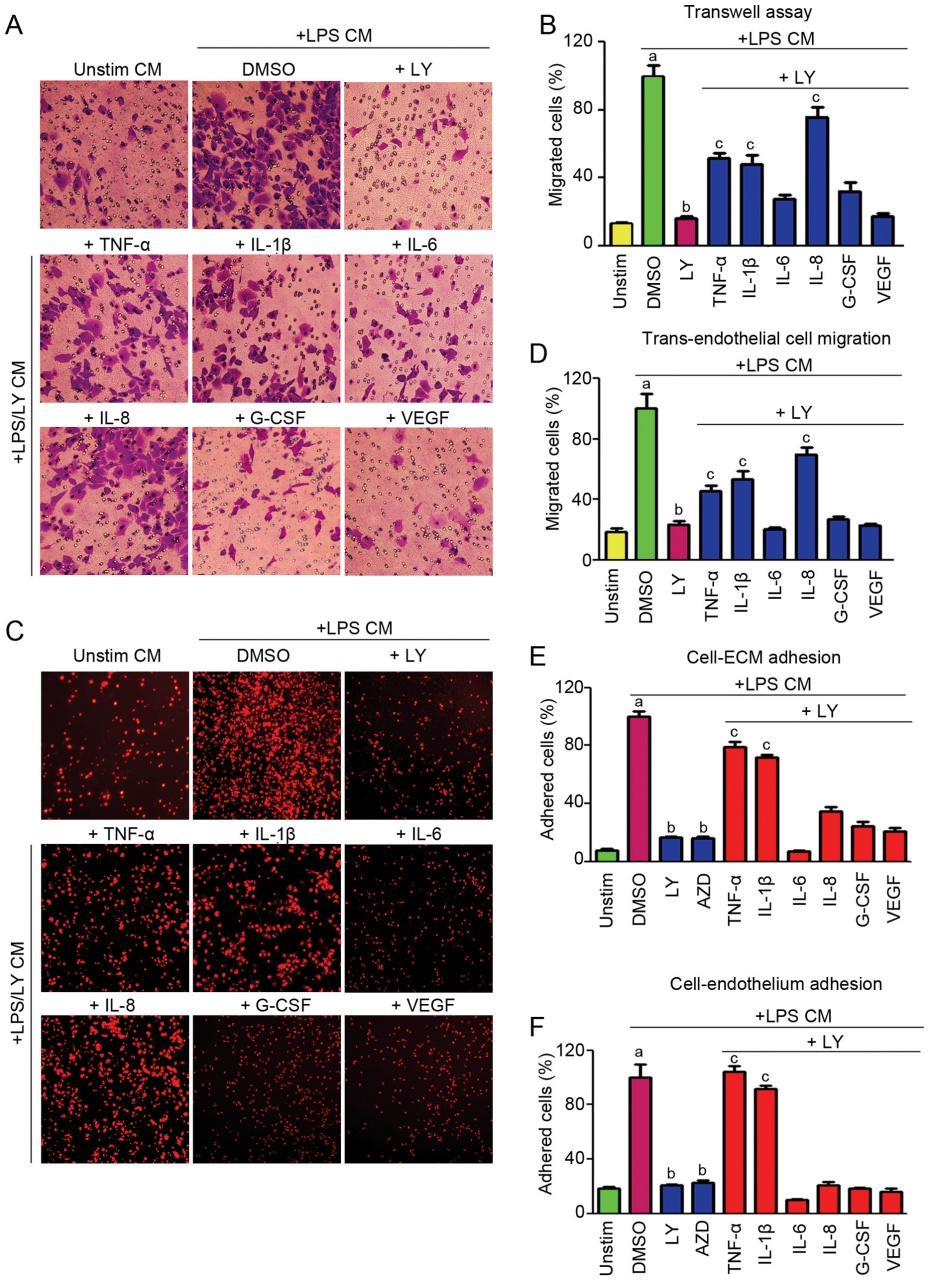
PI3K pathway is involved in motility and adhesion of THP-1 derived macrophages. (A) Examination of the motility of THP-1 derived macrophages with transwell assay, (B) Statistical analysis of the number of migrated cells in panel A. (C) Determination of trans-endothelial cell migration. (D) Statistical analysis of the number of migrated cells in panel C. (E) Determination of the adhesion ability of THP-1 derived macrophages on ECM. (F) Determination of the adhesion ability of THP-1 derived macrophages on endothelium. a indicates P<0.05 versus Unstim. b indicates P<0.05 versus DMSO. c indicates P<0.05 versus LY.

Macrophages are motile and move towards the chemokines to phagocytose the invading pathogens. To examine whether PI3K pathway is involved in the motility of macrophages, we performed transwell assays by seeding THP-1 derived macrophages into the inverts, and the inserts were then placed in a 24-well plate containing conditioned media from THP-1 derived macrophages, which were pretreated with/without LY prior to LPS stimulation. Different proinflammatory cytokines were added to the lower chambers to investigate whether they are involved in cell motility. As shown in Figure 5A, conditioned media without LPS and LY treatment (unstim CM) could not induce macrophages to migrate through the porous membrane. By contrast, LPS treated conditioned media (+LPS CM) notably increased the migration of macrophages, whereas LY neutralized the effect of LPS on migration. However, this neutralization was significantly rescued by IL-8. In addition, TNF-α and IL-1β could partially increase the migration ability of macrophages. In contrast, IL-6 and VEGF had little effect on the migration of THP-1 derived macrophages (Figure 5A and B).

Subsequently, we conducted trans-endothelial cell migration assays to analyze the ability of THP-1 derived macrophages to migrate across the endothelium. The FluorBlok inserts precoated with fibronectin were plated with HUVECs to form the endothelial monolayer, and calcein-AM labeled macrophages were then plated onto the confluent HUVEC monolayer. Migrated macrophages were photographed under the inverted fluorescent microscope, and meanwhile the fluorescent intensities were measured from the bottom of the wells. As shown in the Figure 5C and D, LY treatment attenuated the LPS induced migration, while TNF-α, IL-1β, and IL-8 could restore the trans-endothelial migration, which was restricted by LY treatment.

Cell adhesion is a requisite step for cell motility. In the present study, we examined the interaction of THP-1 derived macrophages with the extracellular matrix (ECM) and endothelial cells. THP-1 derived macrophages labeled with calcein-AM were suspended in different conditioned media with/without cytokines and seeded into wells precoated with fibronectin or HUVECs. The non-adherent cells were washed away and adherent cells were measured at excitation/emission, 485/530 nm. LY and AZD treated conditioned media suppressed the adhesion of macrophages on ECM, which was dramatically enhanced by TNF-α and IL-1β (Figure 5E and F). These data thus indicate that the proinflammatory cytokines mediated by PI3K signaling are involved in macrophage motility and adhesion, which are requisite for the recruitment and infiltration of macrophages into atherosclerotic plaques or inflammatory regions.

### PI3K Signaling is Involved in Neovascularization Associated with its Effect on VEGF Secretion

**Figure 6:**
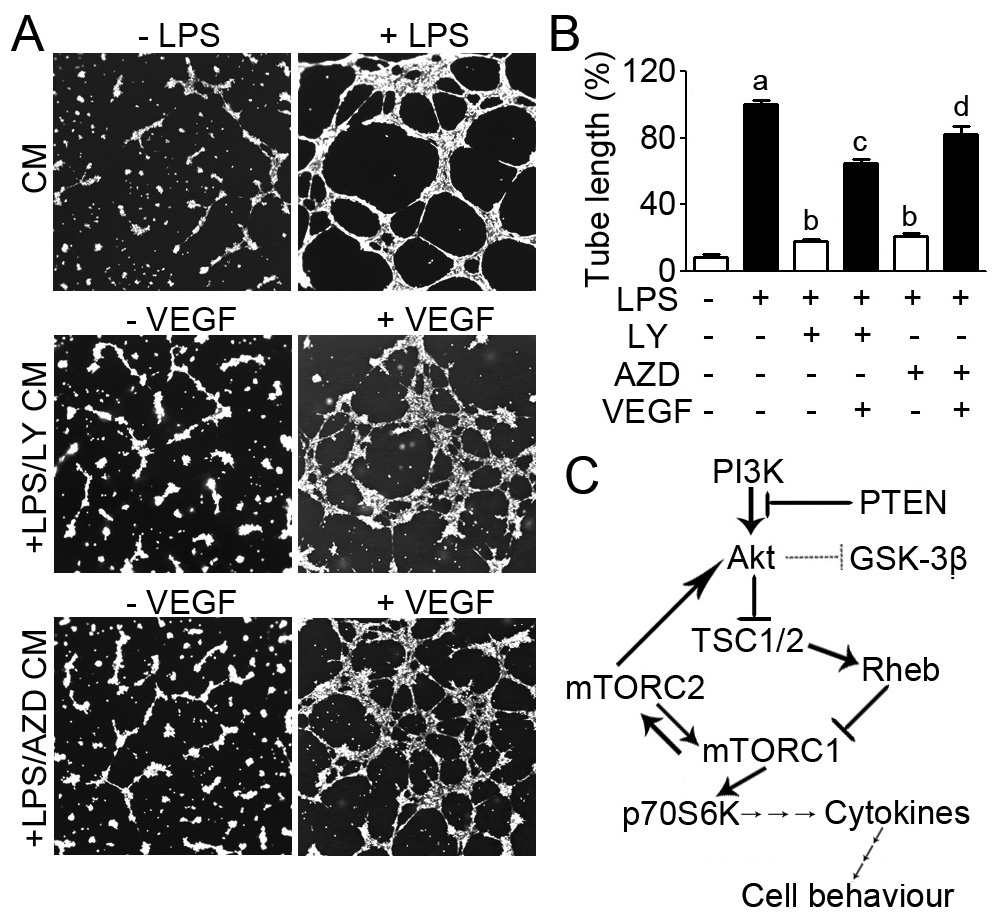
VEGF rescues vascular endothelial tube formation suppressed by PI3K pathway inhibitors. (A) Photographs of the formation of tube-like structures. (B) Experiments were performed as in panel A, and the cumulative tube length was examined. a, b, c, and d indicate P<0.05 versus Unstim, DMSO, LY, and AZD, respectively. (C) Diagram of the PI3K signaling in immune cells.

PI3K signaling inhibitors suppress the production of VEGF, which acts as an angiogenic factor to induce capillary structure formation. To examine the effect of PI3K signaling inhibitors on neovascularization, we conducted tube formation *in vitro* by plating HUVECs onto matrigel to induce the formation of capillary structure. Accordingly, rescue assays were performed by addition of VEGF to investigate whether the role of PI3K signaling in neovascularization is orchestrated through its action on VEGF secretion. As shown in Figure 6A, LPS treated conditioned media induced complete tubular structure formation, whereas both LY and AZD remarkably abrogated the LPS induced tube formation; however, this abrogation was restored by addition of VEGF. The cumulative tube length was measured and shown in Figure 6B. Taken together, these results reveal that PI3K signaling influences neovascularization via its suppression of VEGF secretion from macrophages.

## Discussion

The present study suggests that PI3K regulates the activity of its downstream effectors through AKT/mTOR/p70^S6K^ signaling axis and thus varies the production of cytokines in innate immune cells (Figure 6C). Subsequently, these cytokines exert their roles on cell behaviors such as recruitment of remote immune cells to inflammatory sites and modulation of neovascularization in atherosclerotic plaques (Figure 6C).

Genetic alterations including somatic mutation and amplification of PI3K pathway components are involved in tumor development and occur in many cancer types [24]. Accumulating evidence shows that PI3K signaling is activated in cancer; for example, receptor tyrosine kinases or RAS, which is mediated by oncogenic signaling, can lead to PI3K pathway activation [25]. It is widely accepted that targeting PI3K signaling is an effective approach for cancer therapy. However, few agents targeting this pathway were approved to market despite huge funds were pumped into this field. In this study, we found that PI3K signaling inhibitors significantly influenced the secretion of cytokines from monocytes and macrophages. While it is well documented that cytokines play an important role in anti-cancer immunity, our data provide the underlying explanation for failure of agents targeting PI3K pathway in clinical trials and a rationale for the combined treatment with PI3K/Akt/mTOR inhibitors and immunological therapy.

The PI3K/Akt/mTOR signaling pathway plays a central role in a wide spectrum of cellular activities, including cell proliferation, survival, and differentiation. A growing body of evidence reveals that PI3K pathway is also involved in Toll-like receptor (TLR) signaling and release of cytokines from macrophages [26], [27]. These cytokines could activate several signal pathways, for example, G-CSF regulates the proliferation and release of granulocytes into blood through the Janus kinase/STAT pathway [28]. It is well characterized that PI3K/Akt signaling pathway regulates the inactivation of GSK-3β or activation of mTOR depending on different cell types [29]–[32], contributing to downstream events such as transcription, proliferation, and differentiation. However, the mechanism of PI3K pathway in macrophages is not well characterized. In this study, we found that inhibition of PI3K signaling pathway using PI3K inhibitors LY and Wortmannin, or mTOR inhibitors AZD and Rapamycin mainly results in inactivity of p70^S6K^, other than GSK-3β. Accordingly, both PI3K inhibitors and mTOR inhibitors could remarkably suppressed the secretion of cytokines. These data suggest that PI3K pathway is involved in proinflammatory cytokine secretion through Akt/mTOR/p70^S6K^, and could be a potential target for inflammatory disorders.

Macrophages are long lived immune cells, continually producing proinflammatory cytokines/chemokines and leading to long periods of response to repeated stimulation [33], which is the underlying feature of many chronic inflammatory diseases. Upon inflammatory stimulation, macrophages are activated and differentiated into M1 (classically activated macrophages) and M2 (alternatively activated macrophages) subpopulations [34], [35]. M1 macrophages produce high levels of inflammatory cytokines and chemokines, including IL-1β, IL-6, IL-8, IL-12, and TNF-α. LPS stimulation could polarize THP-1 derived macrophages into M1 subpopulation [36]. In the present study, we apply Luminex and MSD based multiplex technologies to evaluate simultaneously the production of IL-1β, IL-6, IL-8, TNF-α, G-CSF, and VEGF. Luminex xMAP and MSD technologies are powerful tools in immunoassay application with high performance, and the selected proinflammatory panel is reported to be associated with numerous disorders, and could serve as biomarkers for basic research, drug discovery, and disease diagnostics [37]. Our multiplex assays provide accurate and reliable investigation on these pivotal cytokines, which are key mediators of various types of inflammation.

Recruitment of blood-derived macrophages is essential for tissue repair and wound healing. Inflammatory macrophages consisting of the majority of leukocytes, migrate to the peripheral region to respond to the pathogen [38]; however, in pathological conditions, inflammatory macrophages usually lead to progressive tissue damage and autoimmune disease. In the current study, we investigated the effect of conditioned media from THP-1 derived macrophages on macrophage infiltration. Importantly, we found that LY treated conditioned media inhibited the infiltration of macrophages, suggesting that PI3K pathway may mediate the motility of macrophages through the suppression of proinflammatory cytokines/chemokines. To confirm this hypothesis, we added cytokines to examine whether they could restore macrophage migration. Our data demonstrate that the cytokines mediated by PI3K pathway influence the adhesion and motility of macrophages, suggesting PI3K pathway could be a potential target for macrophage-associated disorders.

Numerous studies have demonstrated that infiltration of macrophages plays a key role in atherosclerosis. Macrophages are recruited to the vascular walls to phagocytose oxidized low-density lipoproteins in the initiation of atherosclerosis [39]. In addition to phagocytosis, macrophages secrete a diversity of cytokines including IL-1β, IL-8, and TNF-α, which lead to endothelial dysfunction [40]. Our previous study has demonstrated that neovascularization is a potential target for atherosclerosis [41]. Herein we analyzed the influence of conditioned media of macrophages on neovascularization. Our data show that PI3K pathway inhibitors are involved in neovascularization possibly through modulation of VEGF secretion. Given that macrophage accumulation and plaque neovascularization are main culprits of advanced atherosclerosis [42], our findings provide an important insight for targeting the PI3K pathway as a potential strategy for the treatment of atherosclerosis.

## Supporting Information

Figure S1 **Time-course examination of proinflammatory cytokines secreted by THP-1 monocytes and THP-1 derived macrophages.**
(DOC)
