# Supplementary material for: Identification of a Role for the PI3K/AKT/mTOR Signaling Pathway in Innate Immune Cells
Source: PLoS One. 2014 Apr 9;9(4):e94496. doi: 10.1371/journal.pone.0094496 (PMC3981814; doi:10.1371/journal.pone.0094496)
Supplement: Figure S1 — Time-course examination of proinflammatory cytokines secreted by THP-1 monocytes and THP-1 derived macrophages. (DOC) [file pone.0094496.s001.doc]

**Identification of a role for the PI3K/AKT/mTOR signaling pathway in innate immune cells**

SongboXie, Miao Chen, Bing Yan, Xianfei He, Xiwen Chen*, and Dengwen Li*


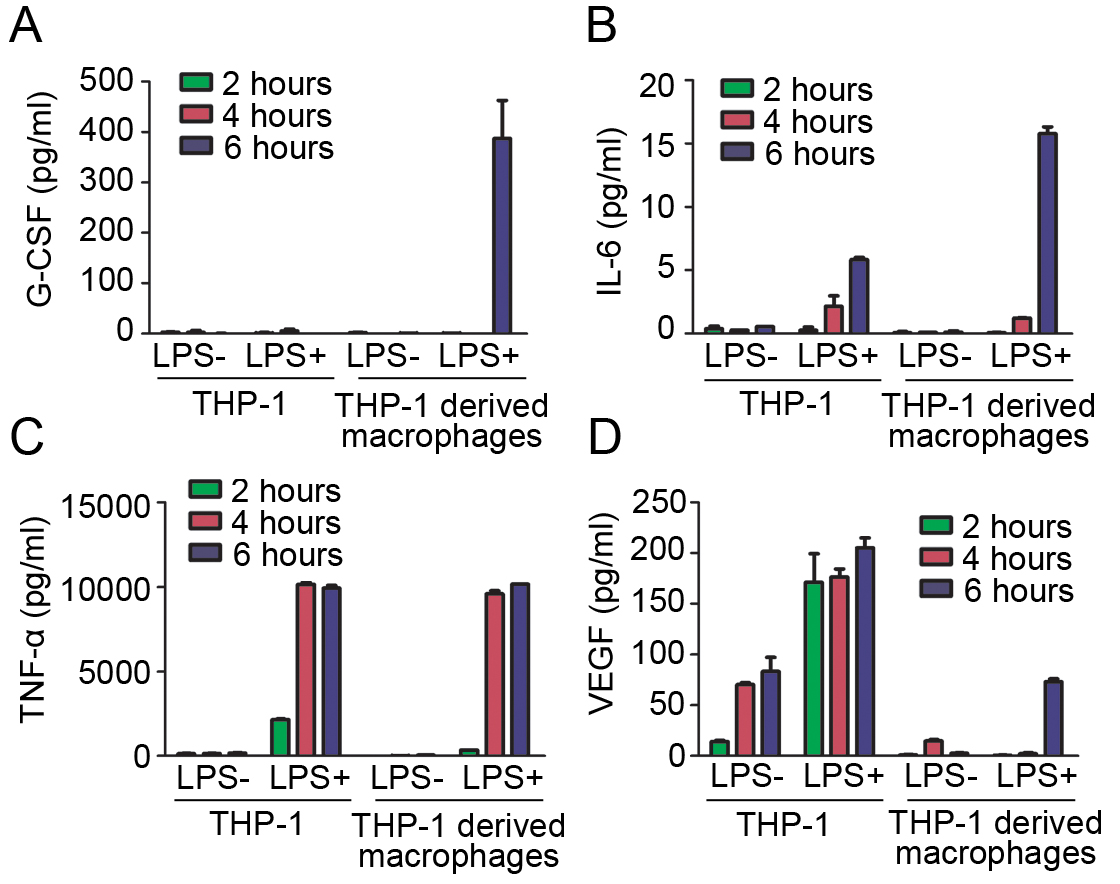


**Supplementary Figure S1. Time-course examination of proinflammatory cytokines secreted by THP-1 monocytes and THP-1 derived macrophages.** (A-D) Analysis of the production of G-CSF, IL-6, TNF-α, and VEGF in supernatants collected 2, 4, and 6 hours of LPS treatment, respectively.
